# Clinical, epidemiological and molecular features of the HIV-1 subtype C and recombinant forms that are circulating in the city of São Paulo, Brazil

**DOI:** 10.1186/1743-422X-9-156

**Published:** 2012-08-09

**Authors:** Rosana Alcalde, Monick L Guimarães, Alberto JS Duarte, Jorge Casseb

**Affiliations:** 1 Laboratory of Dermatology and Immunodeficiencies, Department of Dermatology, Medical School of São Paulo University, LIM56/FMUSP. Av. Dr. Eneas de Carvalho Aguiar, 470, IMT II, 3° andar, 05403-000, São Paulo, SP, Brazil; 2 São Paulo Institute of Tropical Medicine–University of São Paulo, São Paulo, Brazil; 3 Laboratory of Aids and Molecular Immunology, Oswaldo Cruz Institute–FioCruz, Rio de Janeiro, RJ, Brazil

**Keywords:** HIV-1, Subtypes, Recombinants, Resistance, São Paulo, Brazil

## Abstract

**Background:**

The city of Sao Paulo has the highest AIDS case rate, with nearly 60% in Brazil. Despite, several studies involving molecular epidemiology, lack of data regarding a large cohort study has not been published from this city.

**Objectives:**

This study aimed to describe the HIV-1 subtypes, recombinant forms and drug resistance mutations, according to subtype, with emphasis on subtype C and BC recombinants in the city of São Paulo, Brazil.

**Study design:**

RNA was extracted from the plasma samples of 302 HIV-1-seropositive subjects, of which 211 were drug-naive and 82 were exposed to ART. HIV-1 partial *pol* region sequences were used in phylogenetic analyses for subtyping and identification of drug resistance mutations. The envelope gene of subtype C and BC samples was also sequenced.

**Results:**

From partial *pol* gene analyses, 239 samples (79.1%) were assigned as subtype B, 23 (7.6%) were F1, 16 (5.3%) were subtype C and 24 (8%) were mosaics (3 CRF28/CRF29-like). The subtype C and BC recombinants were mainly identified in drug-naïve patients (72.7%) and the heterosexual risk exposure category (86.3%), whereas for subtype B, these values were 69.9% and 57.3%, respectively (p = 0.97 and p = 0.015, respectively). An increasing trend of subtype C and BC recombinants was observed (p < 0.01).

**Conclusion:**

The HIV-1 subtype C and CRFs seem to have emerged over the last few years in the city of São Paulo, principally among the heterosexual population. These findings may have an impact on preventive measures and vaccine development in Brazil.

## Background

The huge genetic variability of HIV-1 results in a complex and dynamic molecular classification of types (HIV-1 and 2), groups (M,N,O,P), and the pandemic group M could be divided into subtypes (A-D,F-H,J,K) and recombinant forms, such as circulating recombinant forms (CRF) and unique recombinant forms (URF). Such HIV-1 variation has an important impact on diagnosis, viral load measurement and the performance of HIV-1 genotyping systems [1-3]. Thus, HIV-1 subtypes also contribute to the capacity of HIV-1 to evade the host immune response, [4] which can affect the response to antiretroviral treatment and, consequently, to the emergence of drug resistance [5]. Some studies have suggested that coreceptor switching from CCR5 to CXCR4 is less common in HIV-1 subtype C, [6] showing a lower rate of accumulation of mutations that confer resistance than subtype B [5]. Concerning the replication fitness of subtype C, the studies were controversial, [7-9] and in relation to transmission in utero, this subtype presented high efficiency compared to subtypes A or D [8,10,11]. These properties suggest an overall transmission advantage, in part, possibly related to efficient replication in dendritic cells, the targets of the onset of HIV-1 infection [11].

Although HIV-1 subtype B is the most studied, HIV-1 C predominates globally and is responsible for approximately 50% of infections [12]. This subtype is not widespread, but it is the most prevalent in sub-Saharan Africa and in the populous countries, such as India and China, where HIV infection rates are highest among heterosexuals [12,13]. Up to now, five circulating recombinant forms (CRFs) involving this subtype have been detected, three of them presenting recombinations between subtypes B and C, CRF07, CRF08, described in China, [14,15] and CRF31_BC, described in Brazil [16]. The other two present recombinations between subtypes C and D, CRF10_CD and CRF41_CD [17,18]. Overall, more than 18% of new infections have been attributed to HIV-1 recombinants [19].

The Brazilian AIDS epidemic is mainly driven by subtypes B, F1, and BF1 recombinants; however, in the Southern region, subtype C and BC recombinants can represent up to 50% of cases, depending of the geographical studied region [20-27]. Despite the documented early circulation of HIV-1 subtype C outside of the southern region in 1992, one case from the city of São Paulo [28] and another in 1986 in Santos, [29] only recently has a continuous increase in cases been observed [30-32].

The current study was conducted to determine HIV-1 genetic diversity and PI/RTI resistance associated mutations among HIV-1 infected drug naïve and HAART patients in a cohort in the city of São Paulo. Moreover, patients classified as HIV-1 subtype C and BC recombinants were analyzed in more details.

## Objectives

The present study aimed to describe HIV-1 subtypes, CRFs and drug resistance mutations in drug-naïve and failing HAART individuals living in the city of São Paulo, in particular attention to those classified as subtype C and BC.

## Study design

### Study population

The study population was composed by HIV-1-infected patients who have been followed-up at the Ambulatory Service of the Department of Secondary Immunodeficiency Clinic of the Clinical Hospital, University of São Paulo Medical School (HC/FMUSP), São Paulo, SP, Brazil, one of the largest teaching and research hospitals in Brazil.

According to the Brazilian Ministry of Health, HIV genotyping test should be routinely performed in patients under antiretroviral therapy who present a plasma viral load over 2000 copies/mL [33]. Following this directive, a total of 82 patients were genotyped at HC/FMUSP from March 2002 to June 2010. During the same period, 211 drug-naive individuals and nine individuals who had unknown status in relation to antiretroviral therapy (ART) were also genotyped. Demographic and clinical data were obtained from clinical charts or direct interview and the patients individually allow us for publication, as shown at Table 1. This study protocol was approved by the Research Ethics Committee of HC/FMUSP, under protocol number 774/99, and written informed consent was obtained from all patients. During the follow-up, CD4^+^T cells and HIV-1 viral load were determined using the Brazilian Ministry of Health guidelines.

**Table 1:** Epidemiological, virological, immunological characteristics and resistance associated mutations of PR/RT region from patients classified as HIV-1

**I.D. LIM56**	**Date collection**	**Age (years)**	**Gender**	**VL**	**CD4**	**Transmission mode**	**Mutations Protease minor**	**Mutations other (Protease)**	**Mutations (NRTI)**	**Mutations (NNRTI)**	**Mutations other (RT)**
**A- subtype C**											
01	22/09/2003	48	M	248000	163	heterosexual	M36I, V82I, I93L	E35D, N37K, R41N, H69K, L89M	None	None	V35T, E36AE, T39E, S48T, K122E, I135T, K166R, F171Y, K173A, Q174K, D177E, I202V, Q207E, R211K, F214L, V245Q
02	25/09/2003	63	M	20500	254	heterosexual	L10I, M36T, I93L	T12P, K14R, I15V, L19I, N37K, R41N, H69K, L89M	None	None	V35T, E36A, T39D, S48T, V60I, K122E, K173A, D177E, T200V, Q207E, R211K
03	18/04/2005	31	M	62900	677	bisexual	M36I, L63P, I93L	T12A, N37K, R41N, H69K, L89M	None	None	V35T, E36A, T39E, K43R, S48T, K122E, D123S, I135T, K173A, D177E, Q207E, R211K, F214L
04	23/05/2005	22	M	17400	528	heterosexual	M36I, I93L	R41K, H69K, L89M	None	None	V35T, E36A, T39E, S48T, V60I, K64R, K122E, D123N, I135T, A158S, K173A, D177Q, Q207E, R211K
05	11/08/2005	37	F	>500000	54	heterosexual	M36I, L63P, I93L	T12A, I15V, L19I, E21EQ, N37K, R41N, H69K	None	None	V35T, T39E, S48T, I135T, K173A, Q174K, D177E, T200A, Q207E, R211K, F214L
06	13/09/2005	37	M	24300	332	heterosexual	M36I, L63P, I93L	N37K, R41N, H69K, L89M	None	None	V35T, E36AE, T39D, K43R, S48T, K122E, D123N, K173A, D177E,V179I, G196E,T200A, E204K, Q207E, R211K
07	10/04/2006	39	F	30500	252	heterosexual	L10V, L63P, I93L	T12AT, I15V, N37K, R41N, H69K	None	None	V35T, E36A, T39D, S48T, K173A, T200A, E204D, Q207AE, R211K
08	11/05/2006	44	M	122000	106	heterosexual	M36I, I93L	I15V, R41K, H69K, T74A, L89M	None	None	K32R, V35T, E36A, T39D, S48T, K173A, Q174K, D177E, I178V, V179I, T200A, Q207E, R211K
09	03/08/2006	23	F	51100	278	heterosexual	M36I, I93L	I13V, I15V, L19I, N37K, R41N, H69K, T74S, L89M	None	K103R, V179D	E28K, K32E, V35T, E36A, T39D, S48T, V60I, S68G, V90I, K122E, D123N, I135T, K173A, D177E, T200A, E203D, Q207A, R211K
10	09/04/2007	25	M	67430	388	homosexual	None	M36T, N37K, R41N, L63P, H69K, L89M, I93L	None	None	V10X, V35T, E36A, T39D, K43R, D121H, K122E, D123S, K173A, D177E, Q197K, Q207K, R211K
11	4/5/2009	30	F	116623	881	heterossexual	L10I	E35D, M36I, N37K, R41N, K45R, D60E, L63Q, H69K, L89M, I93L	None	None	K11R, V35T, E36A, T39D, S48T, K122E, D123N, I135T, K173A, D177E, T200A, E204K, Q207E
12	4/5/2009	29	M	63980	71	heterossexual	None	T12P, I15V, E35D, M36I, N37K, R41N, K45R, D60E, L63Q, H69K, L89M, I93L	None	None	V35T, E36A, T39D, K43KR, S48T, K122E, D123N, I135T, K173A, D177E, T200A, E204K, Q207E, V245Q
13	8/4/2010	31	M	40.557	583	heterossexual	None	I15V, M36I, N37K, R41N, Q61N, L63T, H69K, L89M, I93L	None	K103N	V35T, T39EK, K173A, Q174R, T200A, Q207E, R211K
14	19/4/2010	42	M	59.024	176	heterossexual	T74S	I15V, M36T, N37K, R41N, K45R, L63T, H69K, I72IV, L89IM, I93L	None	None	V35T, E36A, T39E, A158S, K173A, T200A, Q207E, V245Q
15	5/5/2010	47	M	147.776	486	heterossexual	None	T12S, I15IV, L19I, T26AT, M36I, R41K, L63V, H69K	None	None	V35T, E36A, T39E, E40D, K122E, D123S, I135T, S162C, K173A, D177E, T200A, Q207E, R211K, V245Q, E248N
16	6/5/2010	26	F	3.024	289	heterossexual	None	T12S, I15V, L19I, E35D, M36L, N37K, R41N, L63P, H69K, V82I, L89M, I93L	V118I	None	V35T, E36A, T39E, D121H, K122E, D123S, I135T, T165IT, K173T, D177E, I178V, T200A, Q207E, R211K, F214L
**B- subtype BC**											
17	08/03/2005	43	M	5880	353	heterosexual	L63P	I13V, K14R, E35D, I62V, I72V	G333A	None	V35T, E36A, T39D, S48ST, V60IV, K122E, D123S, R211K, V245Q, A272P, I293V, T296S, K311R
18	29/09/2005	40	M	14400	407	heterosexual	L63P	I13V, E35D, I64L, I72V	None	V179D	K22R, V35T, E36A, T39D, S48T, D121H, K122E, D123S, S162C, R211K, V245Q
19	09/01/2006	27	F	96300	345	heterosexual	L63P	I13V, E35D, I64L, I72V	None	V179D	K22R, V35T, E36A, T39D, S48T, D121H, K122E, D123S, R211K, V245Q
20	24/05/2007	42	M	111001	N/C	heterosexual	None	L5F, I13V, L19V, E35D, L63P, I72V, V77I	None	M230W	V35T, E36A, T39D, S48T, K122E, D123S, T165I, R211K, Y232F
21	26/06/2008	44	F	65477	N/C	Others	None	M36I, N37K, R41N, D60E, I62V, L63P, H69K, I93L	M41L, M184V, T215F	K103S, G190A	E28K, K32E, V35T, T39N, V60I, K101Q, K122E, D123N, T200A, V245Q
22	26/4/2010	56	M	38550	250	heterosexual	None	I13V, E35D, L63P, I72V	None	None	V35T, E36A, T39D, S48T, V60I, K122E, D123S, R211K, V245Q

Notes: M: Male; F: Female; VL: RNA HIV viral load; CD4: CD4 T cell count; N/C: data not collected; None: no mutation was found.

### RNA isolation, RT-PCR and DNA sequencing

HIV-1 viral RNA was isolated from plasma samples using the QIAamp® Viral RNA Mini Kit (Qiagen, Hilden, Germany) extraction method, in accordance with the manufacturer’s protocol. Complementary DNA was performed by RT-PCR protocol with random primers and using the PR/RT PCR strategy previously described by Gonzales et al. [34,35]. The PCR products were purified using QIAquick® (Qiagen, Hilden, Germany), in accordance with the manufacturer’s protocol. The PCR purified products were sequenced utilizing ABI Prism Big Dye Terminator Ready Reaction Kit version 3.0 (Applied Biosystems®, Foster City, CA), in accordance with their respective protocols. The reactions were analyzed using the ABI Prism 3100 Genetic Analyzer (Applied Biosystems®, Foster City, CA).

### Sequence analysis

The chromatograms of all sequenced DNA were visually using the Sequencher program version 4.0.5 (Gene Codes) and manually edited. PR/RT sequencing was conducted to detect drug resistance mutations and HIV-1 subtypes. Major antiretroviral drug resistance mutations were classified according to Stanford University online HIV Drug Resistance Database, [36]. The International AIDS Society-USA Panel and Update of the Drug Resistance Mutations in HIV-1, Spring 2008 [37-39].

Nucleotide sequences were aligned using the Clustal X program [40] and later hand edited for minor adjustments and gap-stripped. An alignment of 860 bp that partially covered the PR/RT region (nucleotides 2343-3203) was used for phylogenetic inferences under Neighbor-Joining (NJ) algorithm in Mega 4.0.2 program [41]. In order to confirm the classification of the non-B sequences, Bayesian analysis was performed. The jModeltest 0.1.1 program [42] was used to select the best-fit model of nucleotide substitution under the Akaike information criteria, resulting in the choice of the GTR + I + G model, [43] as implemented in MrBayes v3.1.2 [44]. For each data set, two runs of 4 chains each (one cold and three heated, temp = 0.20) were run for 4x10^7^ generations, with a burn-in of 4x10^6^ generations. Convergence of parameters was assessed by calculating the effective sample size (ESS) using TRACER v1.4 (http://beast.bio.ed.ac.uk/Tracer./) excluding an initial 10% for each run. All parameter estimates for each run showed an ESS values >100. A final Bayesian majority-rule consensus tree was obtained for the data set. The SplitsTree program version 4 [42] was used to confirm the phylogenetic relationship of the recombinant samples using the NeighborNet, based on the pairwise distance estimated by the F84 parameter model, as GTR + I + G model was not available in this program. Recombination analysis was performed by bootscan analysis as implemented in the Simplot version 3.5.1, [44] using group M reference sequences representative of the HIV-1 subtypes. Bootstrap values (>70) supporting branching with reference sequences were determined in NJ trees constructed using the K2-parameter model, [45] based on 100 resamplings, with a 200 nt sliding window moving in steps of 20 bases. In order to confirm the genetic structure of putative recombinant viruses, NJ phylogenetic analyses were conducted using the fragments of sequences assigned to specific HIV-1 subtypes according to the proposed breakpoint position by the bootscanning analysis.

### HIV-1 subtype C and BC *env* sequences

The envelope gene was also sequenced when subtype C and BC PR/RT sequences were identified. A fragment of 0.6 kb of the envelope region of the HIV-1 was amplified by nested-PCR primers LB1 (TAGAATGTACACATGGAATT)/, LB2 (GCCCATAGTGCTTCCTGCTGCT) as outer primers and LB3 (GCAGTCTAGCAGAAGAAGA)/, LB4 (CTTCTCCAATTGTCCCTCATA) as inner primers. The sequence analysis of the *env* region was performed as previously described for the PR/RT region (data not shown).

### Sequence data

All the sequences generated were submitted to the GenBank database and the assigned accession numbers were: *pol* region: GU288708-GU288746, GU288748-GU288754, GU288756-GU288776, GU288778-GU288786, GU288788-GU288792, GU288794-GU288807, GU288809-GU288813, JN195817-JN196018 and *env* region: JN196019-JN196040.

## Results

### Demographic and clinical data

A total of 302 HIV-1-infected patients were analyzed, of these, 225 (75%) were men and 77 (25%) were women, with a mean age of 36 years-old. The distribution by the exposure categories were as follows: 61% heterosexual, 23% men who have sex with men, 9% bisexual and 7% other. According to the clinical status, 153 patients (72.5%) were asymptomatic, 55 (26.1%) were symptomatic and for 3 (1.4%), no information was obtained. The mean RNA plasma viral load was 5.28 log_10_/mL and the CD4^+^T cell count was 350 cells/mm^3^ for naïve patients. For treated patients, 38 (46.3%) were asymptomatic, 27 (33%) were symptomatic and for 17 (20.7%), no information was obtained. The mean RNA plasma viral load was 5.0 log_10_/mL and the CD4^+^T cell count was 246 cells/mm^3^.

### HIV-1 PR/RT subtype classification

**Figure 1:**
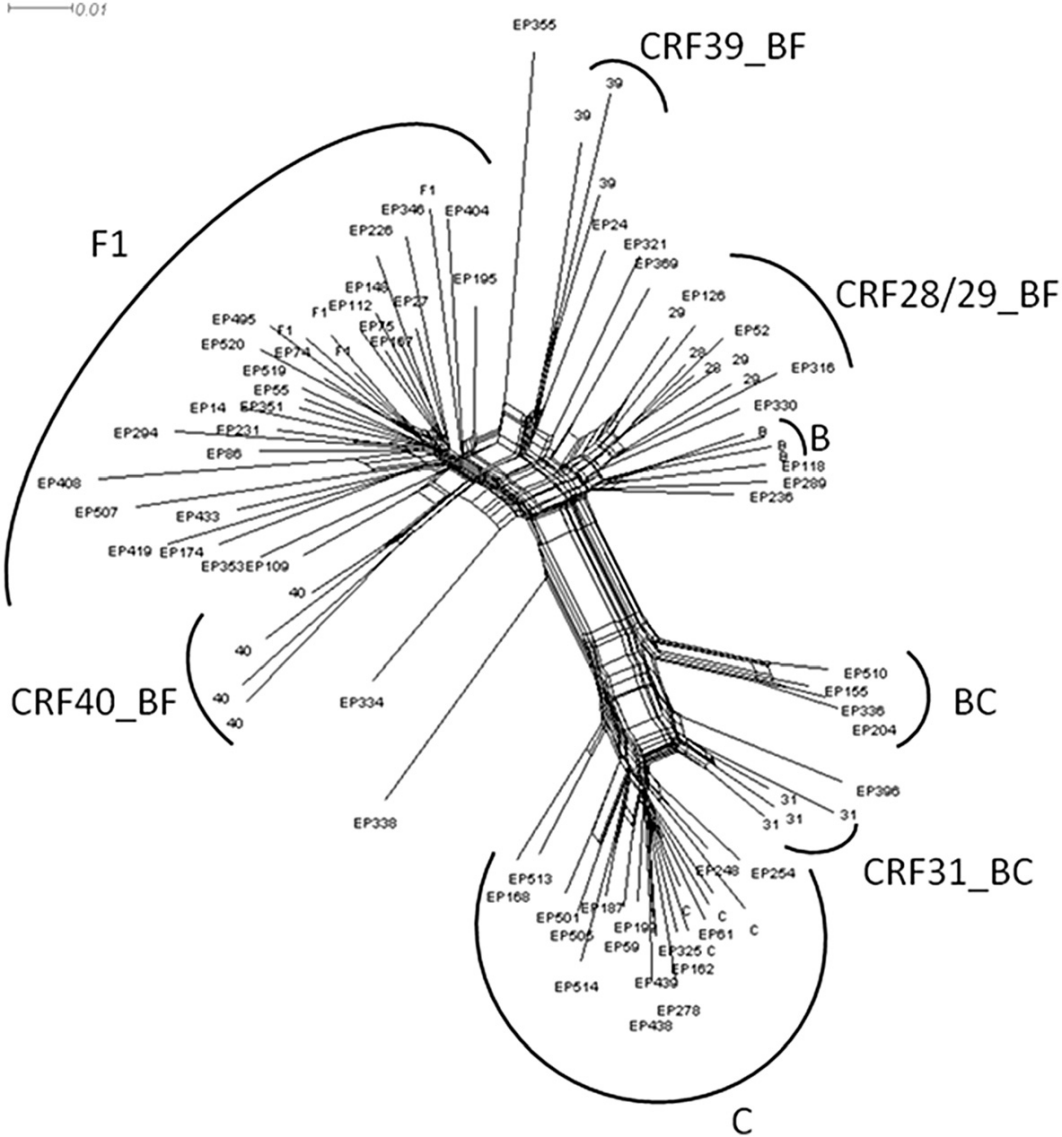
** Majority-rule Bayesian consensus tree of the*pr*/*rt*region (860nt) from non subtype B samples collected in Sao Paulo city from 2002 to 2010.** Posterior probability values superior to 0.80 are indicated. The sequences described in the present study were star marked.

According to the phylogenetic and bootscan analyses, 239 patients (79.1%) were assigned to subtype B (167 naïve, 66 treated and 6 with no information concerning treatment-ND), 23 (7.6%) were assigned to subtype F1 (13 naïve, 8 treated and 2 ND), 16 (5.3%) were subtype C (12 naïve, 3 treated and 1 ND), and 24 (8%) were recombinant forms (19 naïve and 5 treated). Among the recombinant forms, 14 were BF recombinants (11URF_BF1 and 3 CRF28/29), 1 BU, 1 FD, 2 FU and 6 BC recombinants (5 BC with the same PR/RT recombinant patterns, in which only two of them were related and 1 CRF31/D). The Bayesian tree of the non-subtype B sequences is depicted in Figure 1. Interestingly, a group of four BC sequences presenting a well supported clustering and presenting the same recombinant pattern was detected; further full genomic sequencing is required in order to describe a new CRF_BC.

### Primary and secondary resistance

HIV-1 primary resistance mutations were detected in 42 (20%) out of 211 naive individuals, among these, 8 (3.8%) presented major PI resistance mutations, 29 (13.7%) presented NRTI resistance mutations and 27 (12.8%) presented NNRTI resistance mutations. Overall, 20% of individuals presented resistance to one antiretroviral class, 7.6% presented resistance to two classes, and 2.8% presented resistance to all three classes. HIV-1 secondary resistance mutations were investigated in 82 patients receiving HAART regimen, among these, 30 (36.6%) presented major PI resistance mutations, 51 (62.2%) NRTI resistance mutations and 42 (51.2%) NNRTI resistance mutations. Overall, 17.1% of individuals presented resistance to one antiretroviral class, 56.1% presented resistance to two classes, and 76.8% presented resistance to all three classes.

**Figure 2:**
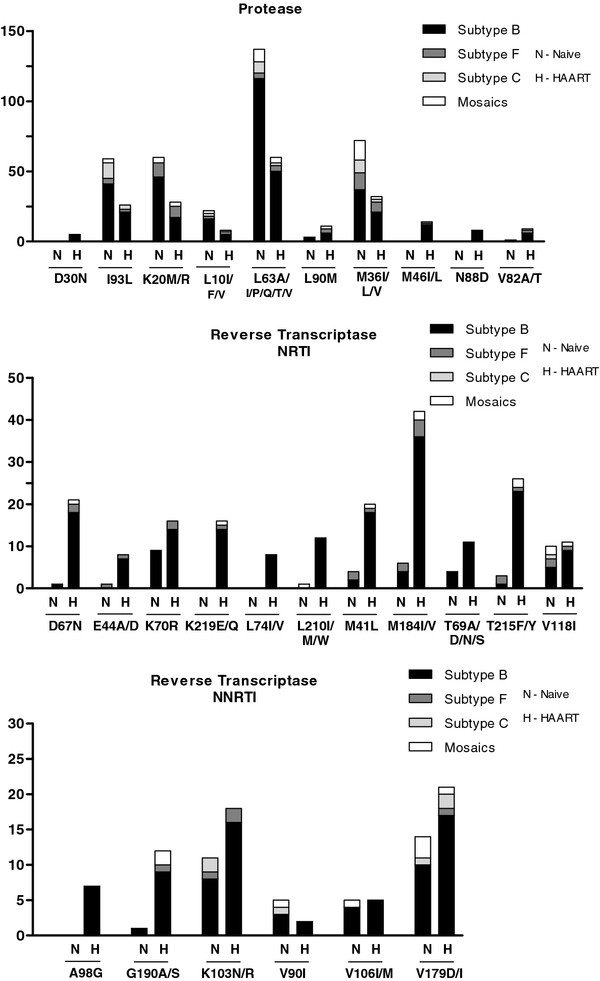
Frequency of the main associated resistance mutations in naïve and HAART patients according to the viral subtype.

Comparing the resistance levels in two distinct periods of the study, 2002–2006 and 2007–2010, the following were verified: PI resistance mutations (5.3% vs 2.6%; p = 0.5), NRTI (12% vs 18%, p = 0.3), NNRTI (10.5% vs 18%; p = 0.18), showing an increasing trend of resistance levels, except for PI resistance mutations. Among the 82 treated patients, the following were verified: PI resistance mutations (46.2% vs 20%; p = 0.03), NRTI (42.3% vs 56.7%; p = 0.3), NNRTI (28.8% vs 46.7%; p = 0.17), similar results to naïve patients were observed (data not shown). The major resistance mutations detected in PR and RT regions in drug naïve or HAART individuals, independent of HIV-1 subtype, were: I93L, L10I/F/V, L63P, M36I/L/V, V118I, V179D/I, as presented in Figure 2.

Secondary resistance mutations in the PR region were detected in individuals infected with subtypes B and F1 (G73S, I50L/V, I54L, I84V, L33F, L89V, V11I, V32I), and in the RT region (K101E, L100I, P225H, V108I, Y181C, Y188L). The mutations K65R, Q151M, Y115F were identified only in subtype B samples.

### Demographical and clinical data from individuals classified as subtype C and BC recombinants

Since very little data is available concerning subtype C individuals outside the southern region of Brazil, in the present work, this group was the focus of more specific analysis. Thus, comparisons between the 22 HIV-1 individuals classified as subtype C and BC recombinants and subtype B individuals verified that the most common transmission mode was sexual and mean age in both groups was approximately 36 years-old. Interestingly, in the subtype C and BC recombinants group, 7 (31.8%) were women and 16 (72.7%) were asymptomatic. The mean viral load and CD4^+^ T cells counts were 4.64 log_10_/mL and 281 cells/mm^3^, respectively, in naive patients, while for patients receiving HAART treatment, the means were 4.23 log_10_/mL and 34 cells/mm^3^, respectively. Among HIV-1-infected individuals in the subtype B group, 47 (19.7%) were women and 155 (64.9%) were asymptomatic (p = 0.97 and p = 0.76; respectively). The means for viral load and CD4^+^ T-cells counts were 5.33 log_10_/mL and 349 cells/mm^3^, respectively, for naive patients, while for patients receiving HAART, the means were 5.02 log_10_/mL and 249 cells/mm^3^, respectively.

An increasing trend of subtype C and BC was observed, increasing from two cases from 2003 to 2006, to 20 cases from 2007 and 2010 (p < 0.05).

Sixteen out of 22 (72.7%) subtype C or BC recombinant individuals were naïve and the main mutations identified in the PR/RT region were G333A, I93L, L63P, M36I/T, V118I. Five (22.7%) were receiving treatment and the main mutations identified were I93L, L63P, M36I/T, M41L, M184V, T215F (to a lesser extent) and for 1 (4.6%) subtype C and BC individual, no information concerning treatment was available. For subtype B individuals, 167 (69.9%) were naïve patients and the main mutations identified were: D67N, K70R, G190A/S, L90M, T69A/D/N/S. Sixty six (27.6%) were receiving treatment and the main mutations identified were A98G, D30N, L74I/V, L210I/M/W, N88D, T69A/D/N/S, V90I, V106I/M, and for 6 (2.5%) individuals, no information concerning treatment was available. These mutations were verified only in the subtype B group.

Only two cases (2/16) showed non-analogue nucleotide drug resistance mutation for subtype C, while four cases (4/6) showed non-analogue nucleotide drugs resistance mutation for subtype BC in naïve patients.

The demographical and clinical data for subtype C and BC recombinant patients with the mutations identified in this cohort are listed in Table 1A and B.

## Discussion

As expect, the HIV-1 B was the most common subtype in the cohort (79.1%) followed by subtype F1 (7.6%). A relevant presence of subtype C (5.3%) was observed and an increase in the number of mosaics (8%), of which 2% were BC recombinants. The first identified case of HIV-1 subtype C in this cohort from the city of São Paulo was from 2003, even though this cohort has been ollowed since 1989. In fact, this subtype predominates in South, where the majority of counties present the highest rates of HIV-1 infection in Brazil, [46] in contrast with Southeastern, where a small incidence of around 3% of subtype C is observed [16,27]. However, the present study and other more recent studies have documented an increased prevalence of this subtype outside of the Southern region [30,47].

The present cohort was predominantly naive heterosexual patients for all HIV-1 subtypes. The mode of transmission for HIV-1 C was clearly associated with heterosexual contact, similar to the major source for this subtype in Africa and Asia [27]. Thus, its presence in the city of São Paulo and the increasing number of cases seem to indicate that subtype C has increased transmission capacity, as occurred in Asia and Africa, the epicenter of the pandemic [30]. It is too early to determine whether the presence of viruses with molecular characteristics of other subtypes has clinical or epidemiological importance, but it certainly indicates the dynamics of the epidemic, with the number of new infections and high prevalence of various subtypes circulating in the Brazilian population.

Recombinations between subtypes B and C have been identified among the HIV-1 samples obtained in Southern Brazil, [22] and now in this cohort. Although subtype C is responsible for more than 56% of HIV-1 infections worldwide, up to now, only four out of the 49 known CRFs involving recombinations with subtype C have been described worldwide: CRF07_BC, CRF08_BC, CRF10_CD and CRF41_CD (CRF41_CD, has not yet been published) [14,15,17]. So far, only one of these is present in Brazil, [18] CRF31_BC [16]. The five BC recombinants identified in this work could represent a new CRF_BC, but full genomic analysis is required to elucidate this possibility.

As previously described, the prevalence of resistance associated mutations appear to differ between subtype B and others (non-B), selected mutations in codons 41, 210 and 215 are more frequent in the former, whereas mutations at codon 67, 70 and 219 are more common in subtype C [31]. The L210W mutation appeared in subtypes B and F, while the mutation L210I/M appeared only in subtype BU. The common resistance associated mutations for all HIV-1 subtypes are 118, 184 and 215. Mutations M36I, L63P, L89M, and I93L are related to antiretroviral resistance in subtype B and have also been identified in subtype C as polymorphisms, [48] these mutations were also verified in patients from the present cohort. In the samples obtained here, the most common resistance associated mutations detected in all HIV-1 subtypes were: PI codons 10, 36, 63 and 93 and NNRTI codons 179 and 190. The K103N mutation was not observed in the mosaics samples and the V90I mutation was not verified in subtype F.

The mutation V106M was previously described as occurring rapidly in subtype C virus, leading to high level of resistance, [47] even though it was not verified in the samples obtained here. This mutation may be a signature in subtype C patients treated with efavirenz and may have the potential to confer high-level multi-NNRTI resistance [19,48]. Differential drug resistance acquisition was verified among subtypes B and C, in which subtype C viruses apparently acquired a lower number of mutations than subtype B for PI and NRTI, but not for NNRTI [7]. As previously described, our group also observed that the proportion of subtype C or BC mosaics among naive cases is significantly higher than among treated individuals, corroborating the hypothesis of more recent introduction of this subtype and recombinants in the Southeastern region [30].

In conclusion, the percentage of subtype C, BC and CRFs could be increasing in the city of São Paulo in recent years. Molecular epidemiological information concerning HIV-1 strains is proving to be important in elucidating the dynamics of HIV spread and the formulation of future vaccine strategies.

## Competing interests

The authors declare that there are no competing interests.

## Authors’ contributions

RA: Drafted the manuscript, analyzed data; MLG: Statistical analysis, phylogenetic analysis, Drafted the manuscript; ADJS: Revised the drafted the manuscript; JC: Designed the study, analyzed the data and drafted the final version of the manuscript. All authors read and approved the final manuscript.
